# Fruit flies are multistable geniuses

**DOI:** 10.1371/journal.pbio.2005429

**Published:** 2018-02-14

**Authors:** Christopher C. Pack, Jamie C. Theobald

**Affiliations:** 1 Department of Neurology & Neurosurgery, McGill University, Montreal, Quebec, Canada; 2 Department of Biological Sciences, Florida International University, Miami, Florida, United States of America

## Abstract

Our sensory systems have evolved to provide us with information about the external world. Such information is useful only insofar as it leads to actions that enhance fitness, and thus, the link between sensation and action has been thoroughly studied in many species. In insects, for example, specific visual stimuli lead to highly stereotyped responses. In contrast, humans can exhibit a wide range of responses to the same stimulus, as occurs most notably in the phenomenon of multistable perception. On this basis, one might think that humans have a fundamentally different way of generating actions from sensory inputs, but Toepfer et al. show that flies show evidence of multistable perception as well. Specifically, when confronted with a sensory stimulus that can yield different motor responses, flies switch from one response to another with temporal dynamics that are similar to those of humans and other animals. This suggests that the mechanisms that give rise to the rich repertoire of sensory experience in humans have correlates in much simpler nervous systems.

The psychologist J. J. Gibson argued that visual perception concerns the detection of “affordances”—specific cues in the sensory environment that suggest particular actions [1]. Gibson was especially fascinated by the use of visual information for navigation, and there are few better examples of this behavior than the fruit fly *Drosophila melanogaster*.

Fruit flies move around by flapping their wings and closely monitoring sensory signals. For example, a fly that inadvertently turns to the right will always see motion to the left, as the image of the world drifts across its retina. This is a perfect example of a Gibsonian affordance, as it implies that the fly can maintain its course by simply steering in such a way as to cancel the coherent motion of the visual field. By this standard, flies are virtuoso navigators—they can often outmaneuver even the most sophisticated human pilots.

This tight link between visual signals and motor actions has made flies an attractive model for understanding how sensory systems work more generally. Beginning in the 1960s, researchers began to perform controlled experiments in which flies were suspended in space via a stiff rod that was glued to their backs [2] while visual motion stimuli were generated by the experimenter. The flies continued happily flapping their wings, producing tiny torques that allowed for precise experimental control of their visual environment and a simple measurement to characterize their responses. Thus, for the first time in 240 million years, the connection between vision and action was broken.

Early implementations of this experimental setup surrounded the fly with mechanical rotating drums imprinted on the inside with visual patterns [2]. These could deliver simple yaw rotating optic flow or be coupled to the flies’ own torque to generate closed-loop visual feedback. Straightforward as the setup was, these experiments, and similar ones in other insects, led to profoundly important discoveries [3]. By controlling the sensory input and measuring the motor output (wing flapping in flies), it became possible to generate mathematical models that captured the functions performed by the intervening neural pathways. That is, for a given sensory input, one could predict with some fidelity the organism’s output, and these models are still in widespread use today [4].

Somewhat surprisingly, the mechanisms that detect visual motion in insects appear to be conserved, or at least highly similar, in humans [4–7]. But it is often assumed that such similarity ends with the basics of motion detection, as the human experience of visual experience is richer and more complex than the simple input-output relationships discovered in flies. Perhaps the most striking example concerns ambiguous stimuli, which can be seen in 2 or more different ways; most people experience these stimuli as changing from one interpretation to another over time, even though the stimulus itself is constant. A familiar case is the Necker cube (Fig 1), which can be seen in 2 or even 3 orientations. Thus, for humans at least, one input can produce a variety of perceptual outputs, and we say in this case that the percept is “multistable.”

**Fig 1:**
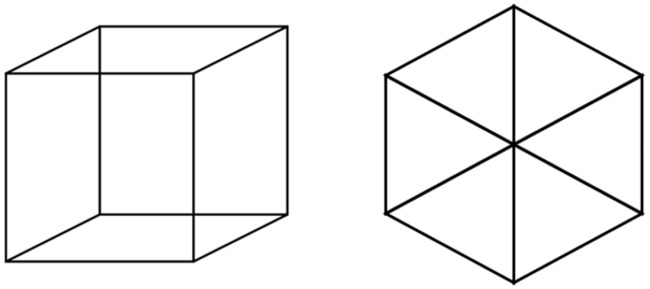
The Necker cube. Staring at the object for several seconds usually leads to switches in the 3-dimensional orientation of the cube. The version on the left has 2 interpretations, while the version on the right has 3.

Multistable percepts have generated profound interest and a fruitful debate regarding their underlying causes. Necker himself attributed the multistability to the mundane influence of eye movements [8], but more recent evidence favors an influence of higher-level cortical functions [9]. Indeed, multistability seems to involve feedback projections from the frontal lobes [9], and it is possible for people to exert voluntary control over the switches in perception [10]. (This can be done by looking at the Necker cube in Fig 1 and imagining one vertex moving forward). As expected from such a cognitive interpretation, multistable percepts manifest themselves differently in people with conditions such as autism [11].

But it remains unknown how widespread these behaviors are in other animals. Many smaller creatures, such as flying insects, have highly limited neural resources at their disposal yet still engage in challenging, visually driven behaviors, such as pursuing mates or landing on targets in a cluttered environment. Insects may in some cases rely on distinct visual algorithms, evolved to function in a small, compact nervous system [12], or they may in other cases implement general visual computations that produce surprising similarities to those of larger animals [13]. Thus, it remains an open question whether insects exhibit anything like multistable perception.

To understand multistability in flies, Toepfer et al. [14] measured the steering efforts of rigidly tethered flying fruit flies while they viewed ambiguous, closed-loop motion patterns. This approach takes advantage of their above-mentioned optomotor response [15], or tendency to minimize retinal slip by following wide-field motion, similar to gaze stabilization in humans [16]. But rather than tracking with just eye movements, flies, which have their eyes immovably fixed in their heads, follow patterns by steering their whole body during flight [17].

Moving beyond the early experimental rigs, a suite of electronic optical arenas have been developed, offering much more varied and flexible image delivery than the old rotating drums [18,19]. Researchers can now generate and deliver almost any imaginable pattern to a flying fruit fly and observe their steering responses.

This includes stimuli that could not be easily produced mechanically, in a lab or natural setting. The authors took advantage of this to test simultaneous conflicting motion stimuli. A fly in closed loop can stabilize a wide-field pattern when it has a bias—in other words, the pattern will drift unless the fly actively steers to stabilize it (Fig 2, top). In nature, this tendency to steady the optic flow helps flies keep on track when they sense self-rotation, perhaps because their wings are damaged, or wind gusts turn them off course. But the authors overlaid 2 stimuli with opposing biases, making both visible and sliding over one another (Fig 2, bottom), which they term the Transparent Panorama Motion Paradigm. This may have some natural analog when insects view the motion parallax of objects at different distances during flight [20], but these patterns generally flow in the same direction, from front to back. When the flows are opposed, as in this experiment, flies can hold steady either moving pattern alone, but combined together, one at most can be steadied and only then by putting the other in fast motion. Flies confronted with confusing patterns sometimes begin steering with reduced connection to the visual stimulus [21].

**Fig 2:**
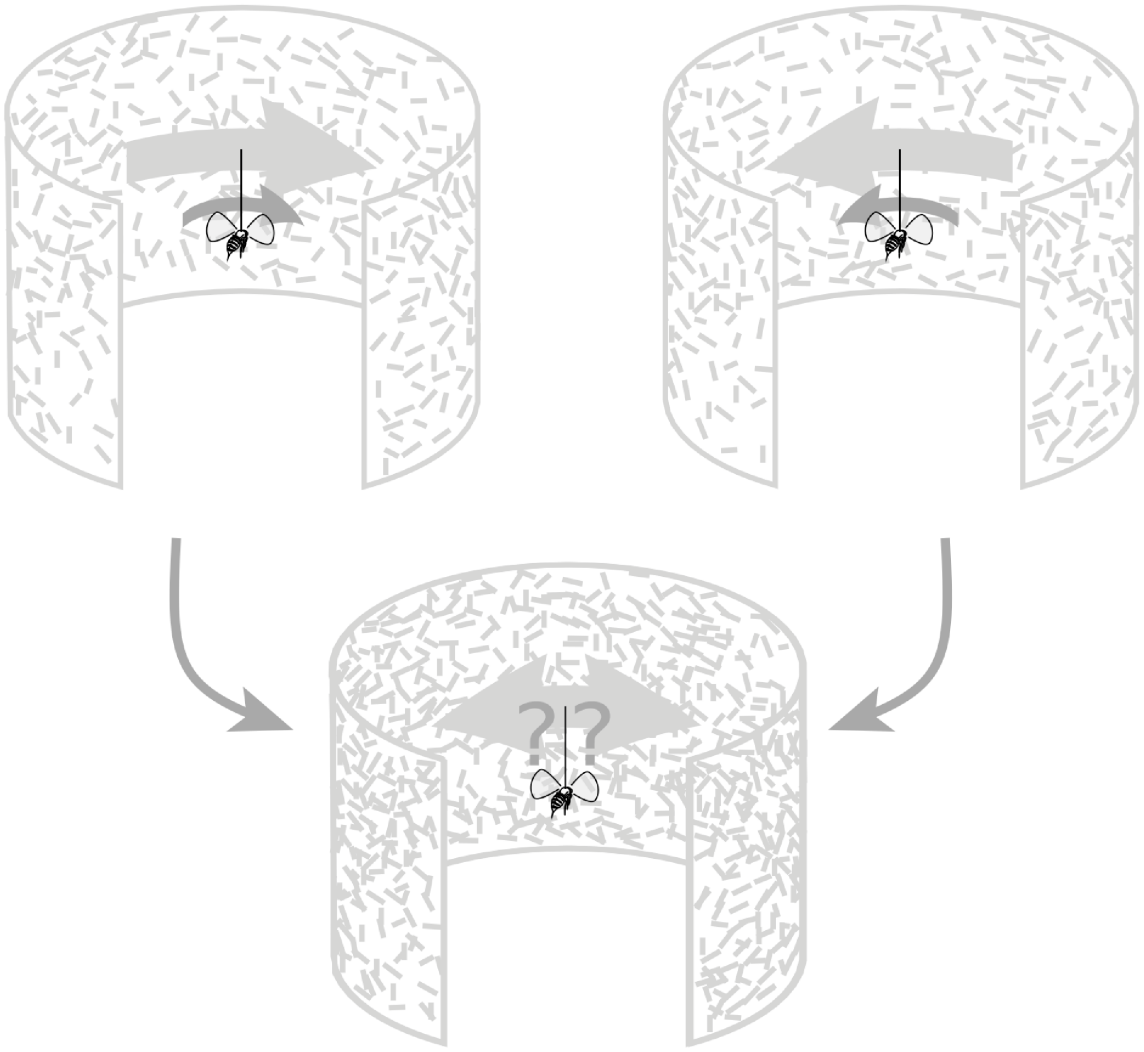
When viewing a drifting pattern, tethered flies respond by attempting to steer with small torques (top), which in a closed-loop feedback arena will steady the pattern. When the patterns and their motions are overlaid, the result is the Transparent Panorama Motion Paradigm for which only some elements can be steadied. Flies respond by switching between a series of tracking behaviors, steadying one pattern, the other, or minimizing the slip of the average of both.

But the overlapping motion-biased patterns generated 3 distinct steering responses from the flies: stabilizing one pattern, the other, or a mean of the 2. In other words, flies shifted between 3 stable states, each of which forced them to tolerate some motion on their retina. This resembles the shifting attention seen in other animals when confronting ambiguous visual stimuli that have multiple possible interpretations.

The experimenters found several stimulus manipulations that alter these stable responses. Intuitively, flies increase the stabilization of one pattern over another when the bias makes it easier to steady. Similarly, when element density increases, pattern stabilization also becomes more common, over steering to the motion average of the patterns. Counterintuitively, however, the authors could increase the time flies spent stabilizing patterns by lowering the contrast, if they lowered the contrast of both patterns. Importantly, this shifting strategy, attending to different elements of a complex pattern, was itself stable over time. The switch between steadying one pattern or the other, or the mean of the 2, continued for extended flight bouts in a seemingly stochastic manner.

These behaviors evoke similarities to classic multistable perception in other animals. Indeed, stimuli similar to those used in the experiment by Toepfer et al. yield a bistable percept in humans: people tend to perceive a cylinder rotating in depth, with the direction of rotation reversing from time to time. Other work in humans has reported multistability with plaid stimuli comprised of 2 gratings that slide over each other [22]. Over time, perception switches from that of transparency (2 separate gratings) to coherence (the tendency to see the gratings as one large pattern). This is conceptually similar to the behavior of the flies in the Transparent Panorama Motion Paradigm. Together, these results imply that, while the phenomenon seems complicated, at least a simple version of it is possible in a much smaller nervous system. The lower complexity and genetic tractability of the fruit fly visual system may make it easier to understand many aspects of multistable perception, such as its relation to figure ground discrimination, a subject of heavy research in flies [23]. As fast flying animals, insects in nature must have the ability to interpret demanding and often ambiguous visual environments to make coherent steering decisions. Their tremendous evolutionary success shows that this property of vision is a basic and important one, even in a very small brain.
